# Transposon Vectors Enable Accelerated Development of Cellular Assay Platforms for the Identification and Characterization of Anti-Viral Small-Molecule Cell-Entry Inhibitors and Neutralizing Antibodies

**DOI:** 10.3390/biomedicines14092119

**Published:** 2026-09-19

**Authors:** Mona Pißarreck, Kristina Katsoutas, Celina Lea Kinder, Jörn Stitz

**Affiliations:** 1 Research Group Medical Biotechnology and Bioengineering, Faculty of Applied Natural Sciences, TH Köln University of Applied Sciences, Campusplatz 1, 51379 Leverkusen, Germany; 2 Institute of Biology, Faculty IV: School of Science and Technology, University of Siegen, 57076 Siegen, Germany

**Keywords:** drug discovery and development, small-molecule inhibitors (SMIs), cell-entry inhibitor, anti-viral, neutralizing antibodies (nAbs), vaccine development, screening platform, virus-neutralization assay, transposon vector, packaging cell line

## Abstract

**Background/Objectives**: Screening for neutralizing antibodies (nAbs) or antiviral small-molecule inhibitors (SMIs) using live virus is laborious and often confined to higher biosafety levels, thus restricting accessibility. The use of murine leukemia virus (MLV)-derived pseudotype vector particles displaying spike or envelope proteins of the targeted human pathogen and susceptible target cells expressing the cognate virus receptor lowers biosafety requirements. Extended drug discovery campaigns demand larger quantities of pseudotype vector particles most favorably produced by stable high-titer suspension vector-producing cells (SVPCs). We aimed to demonstrate that this can be achieved by employing transposon vectors. **Methods**: *Sleeping Beauty* transposon vectors were used to rapidly establish MLV-derived SVPCs using two SARS-CoV-2 spike variants as well as highly susceptible target cells expressing the virus receptor ACE2 and the cellular protease TMPRSS2. Generated cell lines and viral vectors were characterized by employing syncytial-formation assay, Western blot analysis, vector titration and neutralization experiments using two SMIs and two nAbs. **Results**: The expression, fusogenicity and particle incorporation of two spike variants was confirmed and the production of high-titer MLV(SARS-CoV-2) particles was demonstrated. The components of this novel cellular assay platform consisting of stable susceptible target cells and high-yield SVPCs were successfully utilized to characterize nAbs directed against the spike protein and cell-entry-inhibiting SMIs targeting the cellular protease TMPRSS2. **Conclusions**: The proof-of-concept (POC) cellular assay platform components, namely, the two SVPCs and the highly susceptible target cell line, should prove useful in future small-molecule cell-entry inhibitor discovery campaigns, as well as the development of neutralizing antibodies and vaccine candidates directed against SARS-CoV-2. Moreover, the panel of transposon vectors should be easily adaptable to other non-cytotoxic viral pathogens provided these efficiently pseudotype MLV.

## 1. Introduction

A steadily growing number of zoonotic virus infections threatens public health. Examples for human pathogenic viruses include the Ebola virus, Zika virus, influenza virus, human immunodeficiency virus and of course SARS-CoV-2, the infectious agent that caused the COVID-19 pandemic.

SARS-CoV-2 virus particles are decorated with spike protein trimers, and each monomer is structurally divided into a C-terminal membrane-anchored S2 subunit including the fusion peptide and an N-terminal receptor-binding ectodomain S1. The immature, full-length protein is proteolytically cleaved in two places at different stages in the life cycle of the virus, but the subunits remain non-covalently bound in the pre-fusion state [1]. Coronavirus infection occurs through binding of the spike’s receptor binding domain (RBD) located in the S1 subunit to the host cell receptor angiotensin-converting enzyme 2 (ACE2), and fusion is mediated by the activated spike. For activation of spike, processing by the cellular transmembrane serine protease 2 (TMPRSS2) of the S2 subunit at the S2/S2′ site adjacent to the fusion peptide, effectively cleaving off S1 and a fraction of S2 and structural remodeling of the S2 subunit, is key [2]. While human coronaviruses appear to prioritize TMPRSS2-mediated entry, a TMPRSS2-independent endosomal pathway, where available, relies on priming via endosomal cathepsins L/B for fusion with the endosomal membrane [2,3].

One promising therapeutic strategy to fight viral infectious diseases is the development of molecules prohibiting the virus from entering the host cells. To identify virus cell-entry-inhibiting molecules, large compound and antibody libraries, respectively, have to be subjected to multiple screening rounds [4,5,6,7,8,9]. The use of replication-competent wildtype virus isolates or molecular clones of the human pathogen requires handling under bio-safety level 3 (BSL-3) or even level 4 (BSL-4). In addition, quantification of a replicating virus, and thus also its inhibition, is laborious and renders screening campaigns cumbersome and more cost-intensive [10].

To overcome these limitations, replication-incompetent viral vector particles were developed. Most frequently, such pseudotyped vector particles were derived from the lentivirus HIV or the γ-retrovirus murine leukemia virus (MLV) displaying spike or envelope proteins of heterologous viruses such as influenza virus A and B (IVA & IVB), Ebola virus (EBOV), Zika virus (ZIKV), respiratory syncytial virus (RSV), Middle East respiratory syndrome corona virus (MERS-CoV) and HIV-1 [11,12,13,14,15,16,17,18,19]. Generating MLV(SARS-CoV-2) pseudotype particles, Moll et al. impressively demonstrated the suitability of vectors replacing the utilization of infectious virus particles in the characterization of neutralizing activities in reactive blood samples [20]. Of course, the use of pseudotype vector particles is limited to the assessment of cell-entry-inhibiting small-molecule inhibitors (SMIs) and neutralizing antibodies (nAbs) specific to the donor virus of the spike or envelope proteins, as the other components are derived from MLV.

The generation of HIV vector particles is limited to production based on the transient transfection of, e.g., human embryo kidney (HEK) 293T cells due to the cytotoxicity of the viral protease. In contrast, all MLV-based vector components, namely, the packaging construct encompassing the gag/pol genes encoding for the core proteins and all viral enzymes, the transfer vector and the spike protein coding construct, allow for the stable and continuous production of pseudotype vector particles in human host cells at BSL-2. The transfer vector can be equipped with easy-to-detect reporter genes such as egfp, luciferase or lacZ, enabling readily quantifiable reporter expression upon vector-mediated gene transduction into susceptible target cells. However, the production of larger amounts of vector particles demanded in molecule library screens to isolate cell-entry inhibitors is hampered by the two-dimensional cultivation of stable adherent producer cell lines. Conclusively, suspension producer cells would be favorable allowing for the straight-forward scaleup of vector-containing culture volumes using shaker flasks or even bioreactors. Moreover and owing to the higher producer cell density per volumes, superior vector yields can be generated in suspension [21].

To be able to establish novel vector producer cell lines adapting to emerging new virus variants and their spike proteins, rapid cell line development is paramount. We previously reported on the utility of *Sleeping Beauty*-based transposon vector systems in the swift establishment of human producer cell lines outcompeting classic stable plasmid transfection both in speed—in a matter of weeks instead of months—and vector titer [22]. This strategy was also instrumental in the generation of suspension vector producer cells (SVPCs) [23,24]. Briefly, human 293-F suspension host cells were co-transfected with MLV packaging and transfer vector and viral envelope protein constructs, where the terminal inverted repeats (TIRs) of the transposon flanked the transgene expression cassettes. Upon co-expression of mRNA encoding the hyper-active transposase SB100x, the enzyme recognizes target sequences within the TIRs, cuts out the flanked DNA fragments and mediates the stable integration into the host cell’s genome. Using selectable marker genes such as antibiotic resistance genes and subsequent selection pressure, polyclonal SVPCs with multiple copies of all three expression cassettes are enriched, achieving very high vector particle yields [21]. This way, stable SVPCs can be established within about a month.

Seven years after the onset of the COVID-19 pandemic, the search for novel entry-inhibitory small molecules and neutralizing monoclonal antibodies against SARS-CoV-2 is still ongoing [9,25]. Utilizing transposon vector technology in this proof-of-concept (POC) study, we thus demonstrate for the first time the rapid establishment of polyclonal SVPCs producing MLV pseudotype particles displaying different spike variants of SARS-CoV-2. Combined with the generation of a highly susceptible target cell line recombinantly overexpressing the cognate ACE2 receptor and TMPRSS2, the cellular assay platform mimics SARS-CoV-2 infection to facilitate screening of substances exhibiting cell-entry interference. The utility of the platform in future screening campaigns was exemplary demonstrated using two neutralizing antibodies targeting the RBD of the spike proteins. In addition and to investigate if screening of therapeutic small molecules would be feasible as well, two spike-independent inhibitory compounds targeting TMPRSS2 were tested for their capacity to inhibit viral pseudotype vector-mediated reporter gene transduction.

## 2. Materials and Methods

### 2.1. Molecular Cloning

The presented cellular assay platform employs two variants of the SARS-CoV-2 spike protein serving as a model or prototype viral antigen. The variants originate from a Wuhan isolate 1 spike gene encoding spike proteins truncated by 19 terminal amino acids, resulting in a cytoplasmic tail of only 18 residues [26,27]. Besides the truncated wildtype spike gene, a second variant was engineered by introducing two amino acid substitutions, namely, D614G and R682Q. Two partial fragments of the gene were amplified using PCR and a third, synthesized fragment containing both sequence alterations was inserted in between using Gibson Assembly. All primers and oligonucleotides are listed in Table 1.

**Figure 1:**
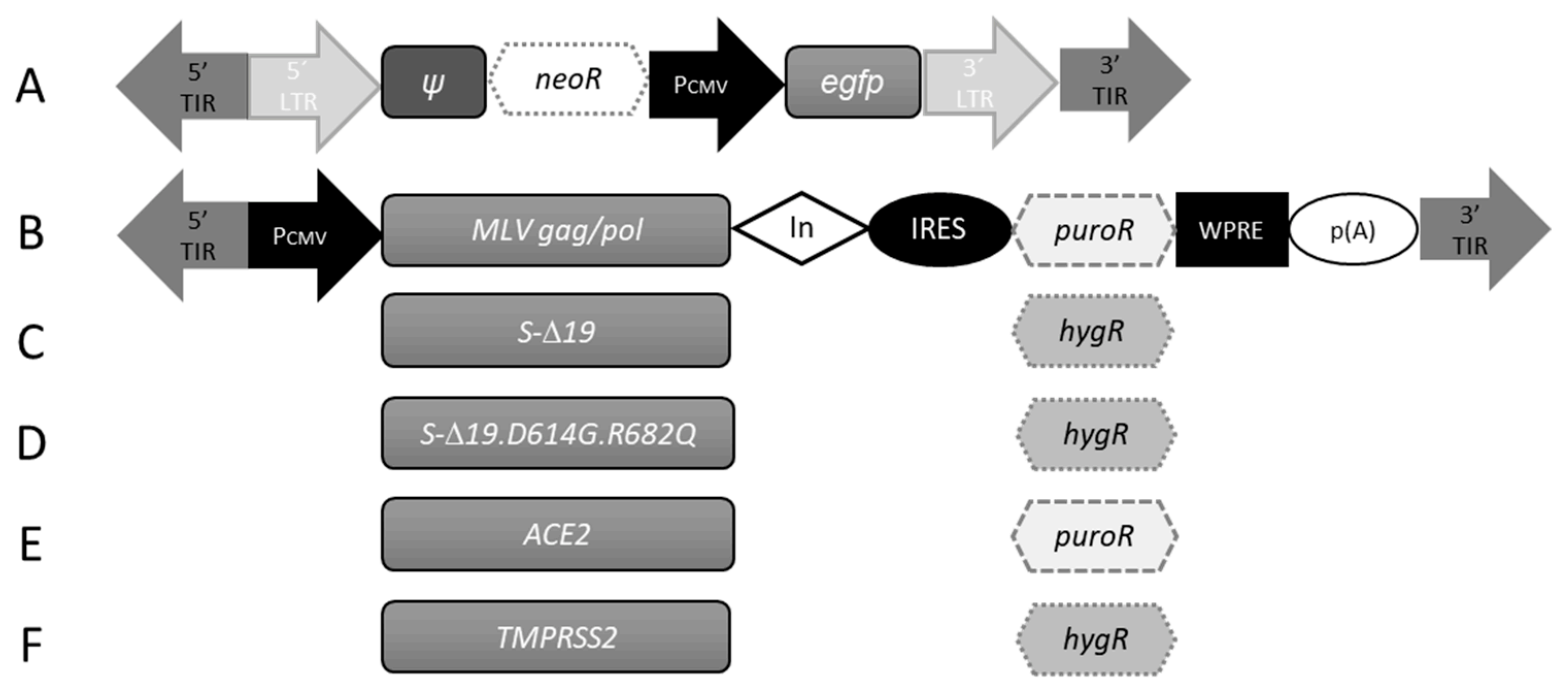
Transposon expression vector constructs. All constructs are framed by terminal inverted repeats (TIRs) of *Sleeping Beauty*. Construct (**A**) shows the transfer vector SB-LEGFP-N1 with MLV long terminal repeats (LTRs) flanking the packaging signal ψ, a neomycin resistance gene (neoR) a CMV promoter/enhancer element (P_CMV_) and the reporter gene efgp. Construct (**B**) depicts the full Sleeping beauty transposon vector cassette used for the following constructs, and only the different transgenes of interest and antibiotic resistance genes are shown in (**C**–**F**). The 5′ TIR and a P_CMV_ driving the expression of the transgene, are followed by the respective transgene, a synthetic intron (In) and an internal ribosome binding site (IRES). The IRES couples the transgene and an antibiotic resistance gene (hygromycn B (hygR) or puromycin (puroR)). A woodchuck hepatitis post-transcriptional regulatory element (WPRE) prolongs the half-life of mRNAs, and a polyadenylation signal (pA) concludes the expression cassette. (**B**) depicts the MLV gag/pol expression cassette, (**C**,**D**) both contain a SARS-CoV-2 spike variant, either with a truncation of the 19 C-terminal amino acids (S-∆19, (**C**)), or by harboring two mutations changing 614D to G and 682R to Q (S-∆19.D614G.R682Q, (**D**)). Construct (**E**) encodes the host cell receptor ACE2 and the expression cassette entailing the cellular protease TMPRSS2 is shown in (**F**).

The first fragment reaches from the very first base of the start codon to the first alteration at 3344 bp. The codon of the 614th residue was altered using primers Spike_fragment 1_FOR and Spike_fragment 1_REV. The use of these primers resulted in a PCR product encoding a part of the spike ectodomain with a 3′-overhang including the intended mutation. The second fragment from the second alteration, R682Q, to the end of the coding sequence, was amplified using the forward primers Spike_fragment 2_FOR and Spike_fragment 2_REV. These two PCR-generated fragments, each carrying one desired codon alteration were fused employing the 279 bp long oligonucleotide Oligo_D614G-R682Q (custom synthesis, Thermo Fisher Scientific, Waltham, MA, USA) using the GeneArt™ Gibson Assembly HiFi Master Mix (Thermo Fisher Scientific, Waltham, MA, USA, # A46628) with a linearized transposon vector to insert ratio of 1:5. As shown in Figure 1C,D, the coding sequences of the truncated SARS-CoV-2 spike-∆19 (S-∆19) and S-∆19.D614G.R682Q were introduced into the expression cassettes of a *Sleeping Beauty* transposon vector by opening the plasmid upon restriction with FastDigest BstXI (Thermo Fisher Scientific, Waltham, MA, USA, # T1120S) and subsequent insertion employing Gibson Assembly utilizing the overhangs generated by primers Spike_fragment 1_FOR and Spike_fragment 2_REV. An ACE2 receptor coding sequence (SinoBiological, Beijing, China; #10108-H05H) and a TMPRSS2 coding sequence, gifted by Roger Reeves (Addgene, Watertown, MA, USA, plasmid #53887; http://n2t.net/addgene:53887 (accessed on 16 September 2026); RRID:Addgene_53887), were inserted in *Sleeping Beauty* transposon vectors as previously described using primers ACE2_FOR and ACE2-REV as well as TMPRSS2_FOR and TMPRSS2_REV, respectively (Figure 1E,F) [28]. Each transgene is coupled to an antibiotic resistance gene within the expression cassette, facilitating the selection of high-level-expressing transfectants using increasing concentrations of the respective antibiotic supplemented to the culture media.

### 2.2. Cells

The generation of human ACE2- and TMPRSS2-expressing cells was described previously [28]. Briefly, human embryonic kidney 293T cells (ATCC, Manassas, VA, USA, #CRL-1658) grown in high-glucose Dulbecco’s Modified Eagle Medium (DMEM, Gibco, Thermo Fisher Scientific, Waltham, MA, USA, #11995065) supplemented with 10% Fetal Bovine Serum (Premium FBS, Gibco, Thermo Fisher Scientific, Waltham, MA, USA, #A5670701) were stably transfected with *Sleeping Beauty* transposon vectors [29] containing genes coding for ACE2 and TMPRSS2 as well as SB100X transposase mRNA out using polyethylenimine (PEI) [30]. TMPRSS2 expression was coupled to a hygromycin B and ACE2 to a puromycin resistance gene. To enable stable integration into the cell’s genome upon transposition, SB100X mRNA was co-transfected in a ratio of 1:2 for plasmid to mRNA [24,30] and transfectants were cultured with increasing concentrations of the respective antibiotics of up to 200 µg/mL hygromycin B and up to 10 µg/mL puromycin (Invivogen, Toulouse, France, #ant-hg-1 and #ant-pr-1).

Stable high-titer SVPCs producing pseudotype MLV(SARS-CoV-2) vector particles were established using a suspension cell line adapted to serum-free media. Human embryonic kidney FreeStyle™ 293-F cells (Invitrogen, Waltham, MA, USA, #R79007) grown in suspension in shake flasks at 137 rpm in FreeStyle™ 293 Expression Medium (Gibco, Thermo Fisher Scientific, Waltham, MA, USA, #12338026) were cultivated at 37 °C in a humidified atmosphere with 8% CO_2_. The fluidlab R-300 (Anvajo, Dresden, Germany) was used to determine cell counts and viabilities. The transfer vector SB-LEGFP-N1 harboring an egfp reporter gene and a neomycin resistance gene, and the transposon vector construct SBgpIpW encoding the MLV gag/pol genes and (Figure 1A,B) [22] were used along with either spike construct. Transfection was carried out using PEI [24]. Briefly, 30 million 293-F cells were seeded in 6 mL media and transfected using a total of 6 µg plasmid DNA with a ratio of 2:1:2 (packaging construct to SARS-CoV-2 spike construct to transfer vector) and 12 µg SB100X mRNA in a ratio of 1:2 for plasmid to mRNA. After 3 h, 9 mL media were added and all media was replaced the following day. Selection using increasing antibiotic concentrations was initiated 4 days post-transfection and continued for four weeks until the maximum doses of 200 µg/mL hygromycin B and G418, as well as 10 µg/mL puromycin were reached.

### 2.3. Syncytia-Formation Assay

Naive 293T cells serving as negative controls as well as 293T stably expressing ACE2 and TMPRSS2 (293T/ACE2/TMPRSS2) were co-cultured with 293T cells transiently co-transfected to express EGFP and spike proteins. Six-well dishes (Nunc, Munich, Germany) seeded with 3.0 × 10^5^ naive 293T cells were co-transfected with an EGFP-encoding plasmid (SB-GFP-IpW [29] and pS-Δ19-IhW and pS-Δ19.D614G.R682Q-IhW, respectively, using TransIT^®^-LT1 Transfection Reagent following the manufacturer’s instructions (Mirus, Madison, WI, USA). One day post-transfection, cells were detached, mixed with either 3.0 × 10^5^ naive 293T and 293T/ACE2/TMPRSS2, respectively, and transferred back into six-well dishes. After three hours, an AXIO Vert.A1 (Zeiss, Oberkochen, Germany) was used for fluorescence microscopic analysis.

### 2.4. Western Blotting of SARS-CoV-2 Pseudotyped MLV Vector Particles

Two days prior to the harvest of supernatant for subsequent Western blot analysis, 293-F/MLV(SARS-CoV-2) SVPCs and naive 293-F cells were seeded at a density of 1 × 10^6^ cells/mL in 20 mL. Following centrifugation of the cell suspension for five minutes at 100 g, the supernatants were filtered using 0.45 µm syringe filters (ROTILABO^®^ PVDF, Carl Roth, Karlsruhe, Germany, #P667.1) and 2 mL of cell-free supernatants (CFSNs) were ultra-centrifuged at 21,630 *g* at 4 °C for 99 min using a MIKRO 200R benchtop centrifuge (Hettich, Tuttlingen, Germany). The pellet was lysed for 30 min on ice using 30 µL Pierce RIPA buffer (Thermo Fisher Scientific, USA, #89900). After adding 10 µL 4X Roti^®^LOAD 2 (Carl Roth, Karlsruhe, Germany, #K930.1) with DTT, samples were incubated for 15 min at 95 °C. Samples stemming from vector-containing CFSNs as well as CFSN from naive 293-F cells serving as a negative control were loaded onto an MP TGX Stain-Free Gel (4–20%, Bio-Rad, Hercules, CA, USA, #4561094) for SDS-PAGE and subsequent transfer to a nitrocellulose membrane (Carl Roth, Karlsruhe, Germany, #200L.1). TBS-T buffer (2 mM Tris-HCl, 15 mM NaCl, 0.05% Tween-20, pH 7.4) supplemented with 2% (*w*/*v*) milk powder (TBS-T/MP) was used for blocking. Rabbit anti-S2 primary polyclonal antibodies (Invitrogen, Waltham, MA, USA, #PA1-41165) diluted in TBS-T/MP with a concentration of 1 µg/mL and secondary chicken anti-rabbit IgG-HRP antibodies (Invitrogen, Waltham, MA, USA, #A15987731-92-03113) diluted 1:1000 in TBS-T/MP were used to detect spike proteins. A Friend murine leukemia virus antiserum produced in goats (ATCC, Manassas, VA, USA, #VR1537AS-Gt) was diluted 1:2000 in TBS-T/MP and used to detect MLV p30, combined with donkey anti-goat-HRP secondary antibodies (abcam, Cambridge, UK, #97110) diluted 1:1000 in TBS-T/MP. The membranes were incubated with the primary antibodies overnight, washed three times with TBS-T and incubated with the secondary HRP-coupled antibodies for one hour. After washing, chemiluminescent signals were detected using SuperSignal™ West Pico PLUS Chemiluminescence-Substrate (Thermo Fisher Scientific, Waltham, MA, USA, #34580) and a ChemiDoc imager (Bio-Rad, Hercules, CA, USA).

### 2.5. MLV(SARS-CoV-2) Vector Titration

Vector titers obtained from MLV(SARS-CoV-2) SVPCs were assessed using 293T and 293T/ACE2/TMPRSS2 target cell lines. In a shaker flask, 4 × 10^6^ cells/mL were seeded in 25 mL media. The following day, 100,000 naive 293T and 293T/ACE2/TMPRSS2 cells were seeded per well in 12 well dishes (12-Well CytoOne Plate, Starlab, Hamburg, Germany, #CC7682-7512) and subjected to transduction three hours later. Media was removed from target cells and replaced using undiluted CFSNs as well as different dilutions thereof. Two days post-transduction, the proportion of transduced, EGFP-positive cells was assessed using an SH800s cell sorter (Sony Biotechnology, San Jose, CA, USA) and the FlowJo (v10.9.0_CL) software. Titers were calculated as described by Salmon and Trono [31] and shown in transducing units per mL (TU/mL). Batches of CFSNs were harvested, aliquoted and promptly stored at −80 °C. Three aliquots of the same batch for each pseudotype vector were thawed and used for titration to determine the average titer of the freeze-thawed samples. The software GrapPad Prism 6 was used to analyze data using the unpaired, non-parametric Mann–Whitney test.

### 2.6. Assessment of Transduction Using Small-Molecule Cell-Entry Inhibitors and Neutralizing Antibodies

For both assays described below, highly susceptible 293T/ACE2/TMPRSS2 target cells were used. Cells were seeded as described above and the same volume of thawed CFSNs equivalent to a multiplicity of infection (MOI) of 0.1 was used for all experiments. The only variable were different concentrations of interfering compounds, namely, nAbs and protease inhibitors. The half-maximal inhibitory concentrations (IC50 values) were calculated using GraphPad Prism 6 using a 4-parameter dose–response fit with a 95% confidence interval (CI). Significant difference was determined using an unpaired one-way ANOVA with a post hoc Tukey’s Honestly Significant Difference test with ns = *p* > 0.05, * = *p* < 0.05, ** = *p* < 0.01, *** = *p* < 0.001, **** = *p* < 0.0001 compared to the untreated positive control.

#### 2.6.1. Virus Neutralization Assay (VNA)

Prior to transduction, MLV(SARS-CoV-2) vector particles and different concentrations of nAbs were incubated in a total volume of 50 µL at 8 °C for one hour. After this neutralization step, the samples were diluted using 450 µL media and incubated with 1 × 10^5^ 293T/ACE2/TMPRSS2 cells; 0.5 mL media was added the next day. Two days post-transduction, the efficiency of gene transfer was assessed in titration experiments. Titers were normalized against control samples in the absence of nAbs. The nAbs used in this study were commercially available monoclonal antibodies (mAbs) targeting the RBD of spike, namely, recombinant rabbit mAb HL1003 (Invitrogen, Thermo Fisher Scientific, #MA5-46917) directed against the RBD of the SARS-CoV-2 spike protein (strain Wuhan-Hu-1) and recombinant human anti-RBD mAb P06Dhu (Invitrogen, Thermo Fisher Scientific, #703973).

#### 2.6.2. Small-Molecule Cell-Entry Inhibition Assay

In contrast to the VNA protocol and instead of the vector particles, 293T/ACE2/TMPRSS2 cells were incubated with small-molecule cell-entry inhibitors. The protease inhibitors Nafamostat (MedChemExpress, Monmouth Junction, NJ, USA, #naive-B0190A) and Camostat mesylate (BLDpharm, Shanghai, China, #BD1414) were used to inhibit TMPRSS2 prior to the cells’ exposure to MLV(SARS-CoV-2) vector particles. The inhibitors were initially dissolved in DMSO, with a concentration of 10 mM. A 1 mM working stock was prepared using PBS and further dilutions were prepared using cultivation media. Besides a control containing only media, a DMSO control was included using the same DMSO concentration employed in the highest inhibitor dose. Cells were incubated with 475 µL of the respective solutions for one hour, followed by the addition of 25 µL media containing MLV(SARS-CoV-2) vector particles corresponding to an MOI of 0.1. The next day, we added 0.5 mL media; transduction efficiencies were assessed two days post-transduction and normalized to the cognate controls containing neither inhibitor nor DMSO.

## 3. Results

### 3.1. Both Spike Proteins Are Cell-Surface-Expressed and Show Fusogenicity

To demonstrate this assay’s applicability to different variants of viral antigens, two C-terminally truncated spike variants were used: (i) The Wuhan isolate 1 spike protein (S-∆19) and (ii) a modified variant thereof containing two amino acid substitutions (S-∆19.D614G.R682Q) shown to affect the protein’s stability and processing into the subunits S1 and S2 by cellular furin-like proteases. The D614G mutation was first detected in 2020 and spread rapidly across the globe [32]. The altered residue is located between the RBD and the S1/S2 furin-like cleavage site and is associated with increased infectivity owing to reduced S1-shedding and increased particle decoration [33]. The R682Q mutation, observed when replicating SARS-CoV-2 in Vero E6 cells [34], impairs the S1/S2 cleavage site. This is associated with the loss of fusogenicity and reduced cell entry efficiency via membrane fusion [35]. However, this derogation was shown to be restored in the presence of TMPRSS2 overexpression on the target host cell surface [36]. Both mutations were previously shown to enhance SARS-CoV-2 spike pseudotyping of lentiviral and MLV-derived vector particles [37].

To compare the spike variants S-∆19 and S-∆19.D614G.R682Q, the cognate expression constructs were co-transfected together with an EGFP-encoding reporter plasmid into 293T cells. To assess the spike variants’ surface expression, the ACE2 receptor recruitment, TMPRSS2-mediated priming and subsequent membrane fusion, the transfectants were co-cultivated with naive 293T and recombinant 293T/ACE2/TMPRSS2 cells.

**Figure 2:**
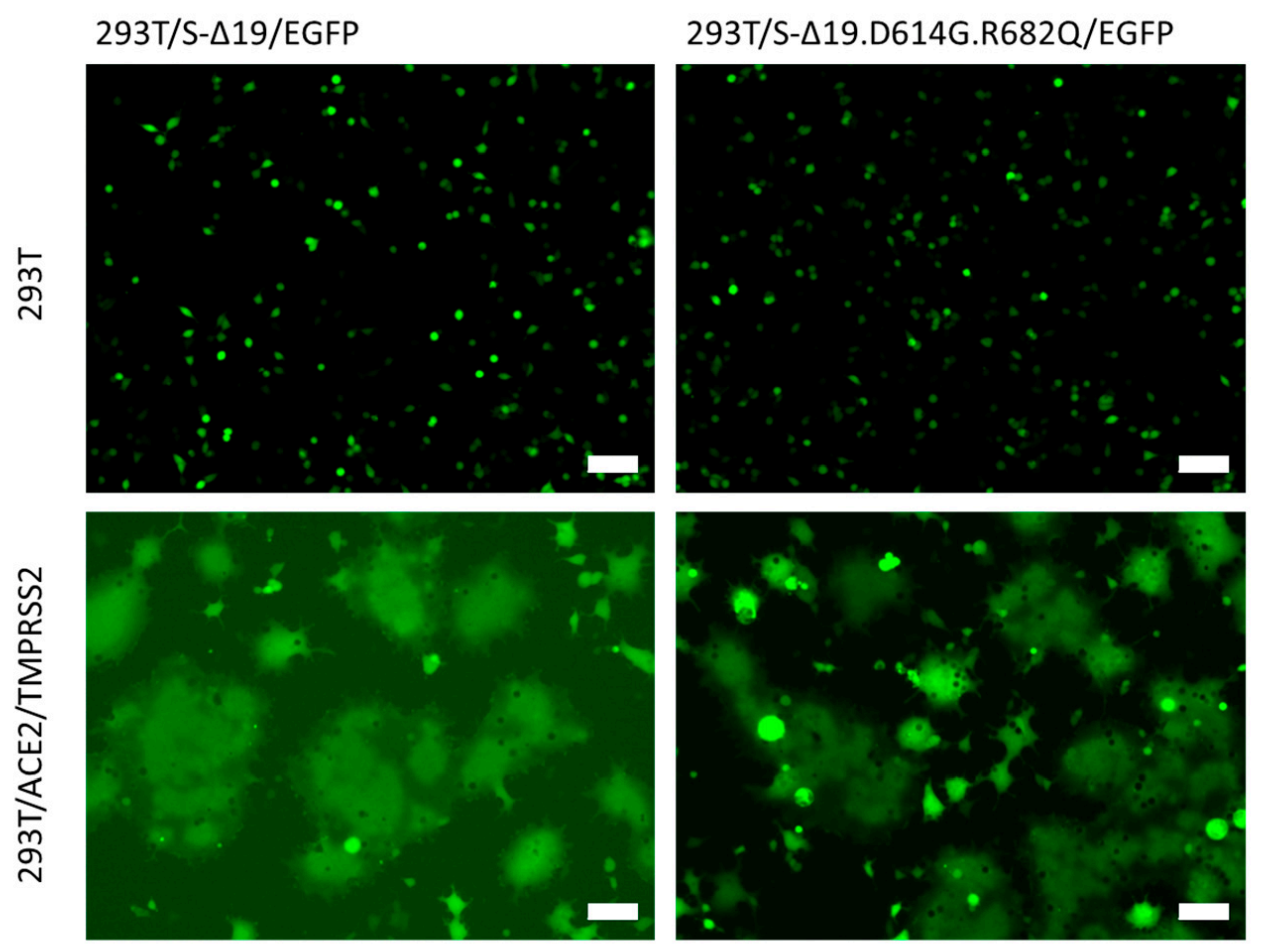
Syncytia formation assay of spike/EGFP-expressing and susceptible target cells to confirm full functionality of both spike variants S-∆19 and S-∆19.D614G.R682Q. Each image represents a co-culture experiment mixing naive 293T and 293T/ACE2/TMPRSS2 cells (rows) with 293T cells transiently expressing EGFP and either S-∆19 (**left**) or S-∆19.D614G.R682Q (**right**). The white scale bar represents 100 µm.

As shown in Figure 2 and already three hours after co-cultivation, syncytia were readily observed in the presence of the 293T/ACE2/TMPRSS2 cell line. In contrast, polynucleated syncytia resulting from the fusion of multiple cells, were not detected in co-cultures of naive 293T, with either 293T/S-∆19 or 293T/S-∆19.D614G.R682Q cells serving as negative controls. As cells expressing S-Δ19, 293T/S-∆19.D614G.R682Q cells resulted in many, yet smaller syncytia indicating a slightly lower fusogenicity. However, both variants were clearly shown to be cell-surface-expressed, binding the cognate receptor and be primed by the cellular membrane-bound protease TMPRSS2.

### 3.2. Spike Variants Are Incorporated into MLV Gag-Formed Particles

293-F suspension cells were co-transfected with the respective spike expression construct, the MLV packaging construct, the MLV-derived transfer vector carrying a CMV promoter driven egfp reporter gene and *Sleeping Beauty* 100X mRNA transcripts to facilitate high copy insertions of the transposon vectors into the host cell genomes. The genotype–phenotype coupling allowed for selection of transgene-positive cells using the respective antibiotics puromycin, G418, and hygromycin B.

**Figure 3:**
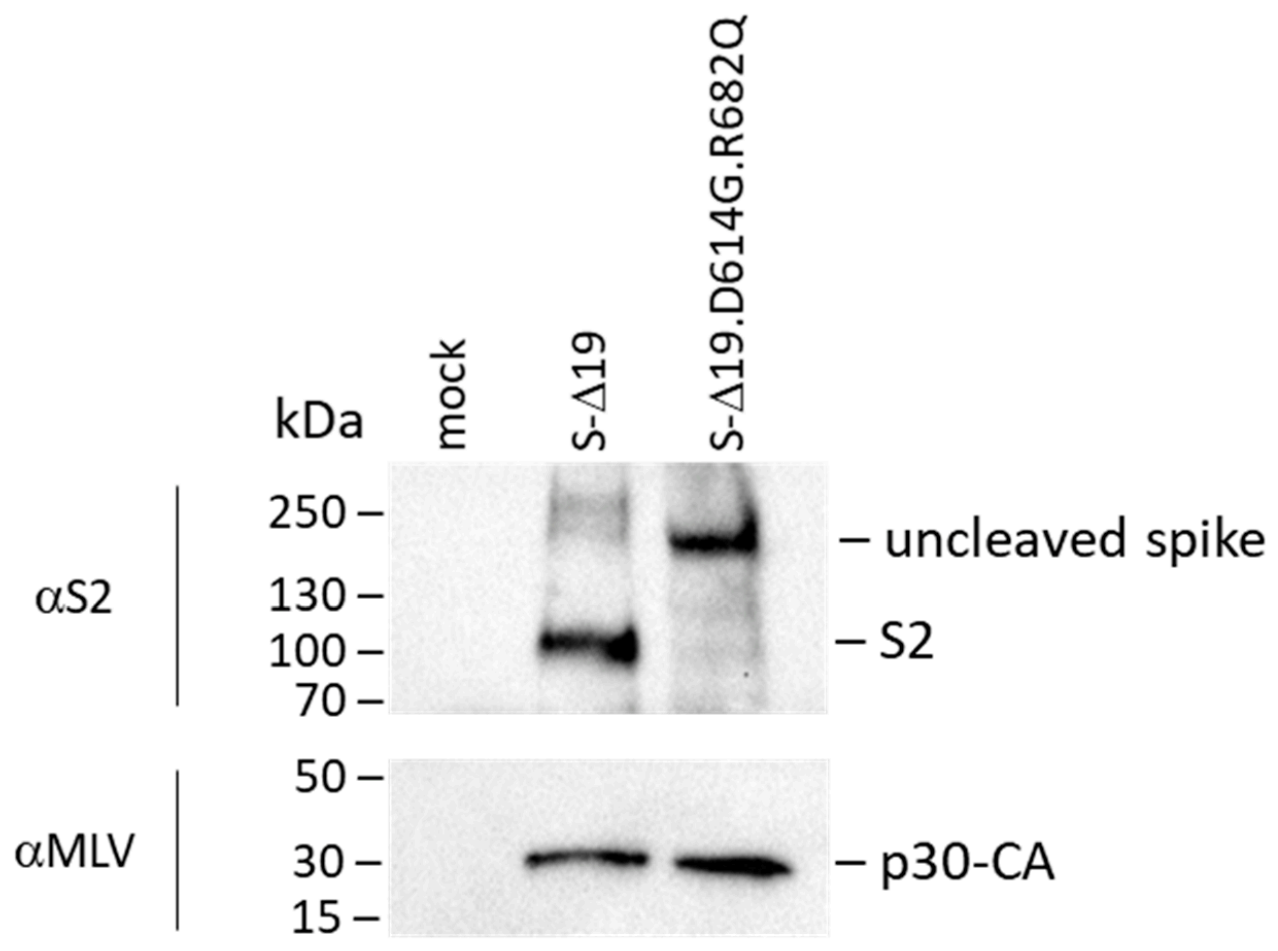
Western blot analysis of MLV(SARS-CoV-2) pseudotype vector particles produced by stable 293-F-derived SVPCs. CFSN from naive 293-F was used as negative control (mock). Polyclonal antibodies specifically targeting the S2 subunit of the SARS-CoV-2 spike protein were employed to detect incorporated spike variants (**upper panel**; uncleaved and processed S2). Polyclonal goat anti-MLV antibodies were used to detect the capsid protein p30-CA (**lower panel**). Respective secondary, HRP-coupled antibodies were used for visualization. No signals were detected in the mock sample. The images presented result from one membrane, which was cut just below the 70 kDa molecular weight marker protein after blotting and processed separately.

To confirm MLV pseudotype vector particle-formation, CFSNs were collected from the SVPCs 293-F/MLV(S-∆19) and 293-F/MLV(S-∆19.D614G.R682Q) and ultracentrifuged. Pellets were resuspended and subjected to a Western blot analysis, shown in Figure 3. CFSNs from both SVPCs as well as from naive 293-F cells (mock) serving as a negative control were analyzed using polyclonal antibodies directed against the S2 subunit of the SARS-CoV-2 spike and polyclonal antibodies targeting MLV, respectively, along with the cognate secondary antibodies to detect spike proteins and the capsid protein p30-CA.

Neither p30-CA nor spike protein were detected in the mock control. Comparable amounts of p30-CA were detected in both vector preparations indicating similar amounts of particles produced by the SVPCs. Spike proteins were also detected in both samples. As expected, variant S-∆19 showed large quantities of a molecular weight of about 100 kDa corresponding to the processed S2 subunit while only weak signals of the presumably unprocessed precursor were detected at approximately 200 kDa. In contrast, S-∆19.D614G.R682Q revealed primarily the full-length, uncleaved precursor protein.

### 3.3. Titration of Pseudotyped MLV Vector Particles Reveals Titers of More than 1.0 × 10^6^ TU/mL

Transduction experiments were conducted to assess the productivity of the established MLV-derived pseudotype vector-producing cells. In three independent experiments SVPCs were seeded at a high cell density and CFSNs were collected the following day. Different target cells, namely, naive 293T and 293T/ACE2/TMPRSS2 were seeded few hours prior to transduction. Cells were incubated with several dilutions of vector-containing CFSNs in culture media and expanded for two days. Titers were determined by detecting egfp-transgene expression using a flow cytometer. As 293T target cells revealed only very weak gene transfer efficiencies of just over 1.0 × 10^2^ TU/mL in initial experiments, further titrations were conducted exclusively using the novel recombinant and highly susceptible 293T/ACE2/TMPRSS2 cells. As shown in Table 2A, comparable titers of up to 1.52 × 10^6^ and 1.34 × 10^6^ TU/mL were achieved using freshly harvested MLV(S-∆19) and MLV(S-∆19.D614G.R682Q) vector particles, respectively. To assess the loss of functional titers after freezing and thawing, three individual aliquots of one batch of vector-containing CFSNs were used for titration on 293T/ACE2/TMPRSS2 cells (Table 2B). Again, functional vector titers were comparable between MLV(S-∆19) and MLV(S-∆19.D614G.R682Q) but about five-fold lower. Differences between MLV(S-Δ19) and MLV(S-∆19.D614G.R682Q) were not statistically significant (*p* = 0.7 and *p* > 0.999 for titers resulting from freshly harvested and freeze-thawed samples, respectively). The frozen vector samples and detected titers were used in the following virus-neutralization assay.

In a first scale-up experiment, 300 mL of suspensions at densities of 5.0 × 10^6^ 293-F/MLV(S-∆19.D614G.R682Q) cells per mL were cultivated in 1 L shaker flasks. At the point of vector harvest, producer cells showed high viabilities > 90% and similar titers of over 1.0 × 10^6^ TU/mL were achieved.

### 3.4. Antibody-Mediated Neutralization Shows Different Potencies on the Two Pseudotype Vectors

The pseudotype vector particles and highly susceptible cells were tested as components of a virus-neutralization assay (VNA) using two commercially available antibodies directed against the RBD of spike exemplarily. Neutralizing antibodies targeting the RBD at least partially block the receptor binding site, thus preventing receptor binding. All experiments were conducted employing an MOI of 0.1. The only variable was the concentration of monoclonal nAbs rabbit HL1003 and human P06Dhu. The neutralization was carried out prior to transduction at 8 °C for one hour to replace the previous culture media of the seeded target cells. Light-microscopic analyses were conducted daily not revealing any indications of potential cytotoxic activities mediated by the antibodies. Two days post-transduction, cells were analyzed for EGFP expression using flow cytometry. The transduction efficiencies of the positive control (PC)—absence of nAbs during incubation at 8 °C—was set as 100% and those of the neutralized samples were normalized against it. All neutralization assays were performed in three individual transduction experiments.

**Figure 4:**
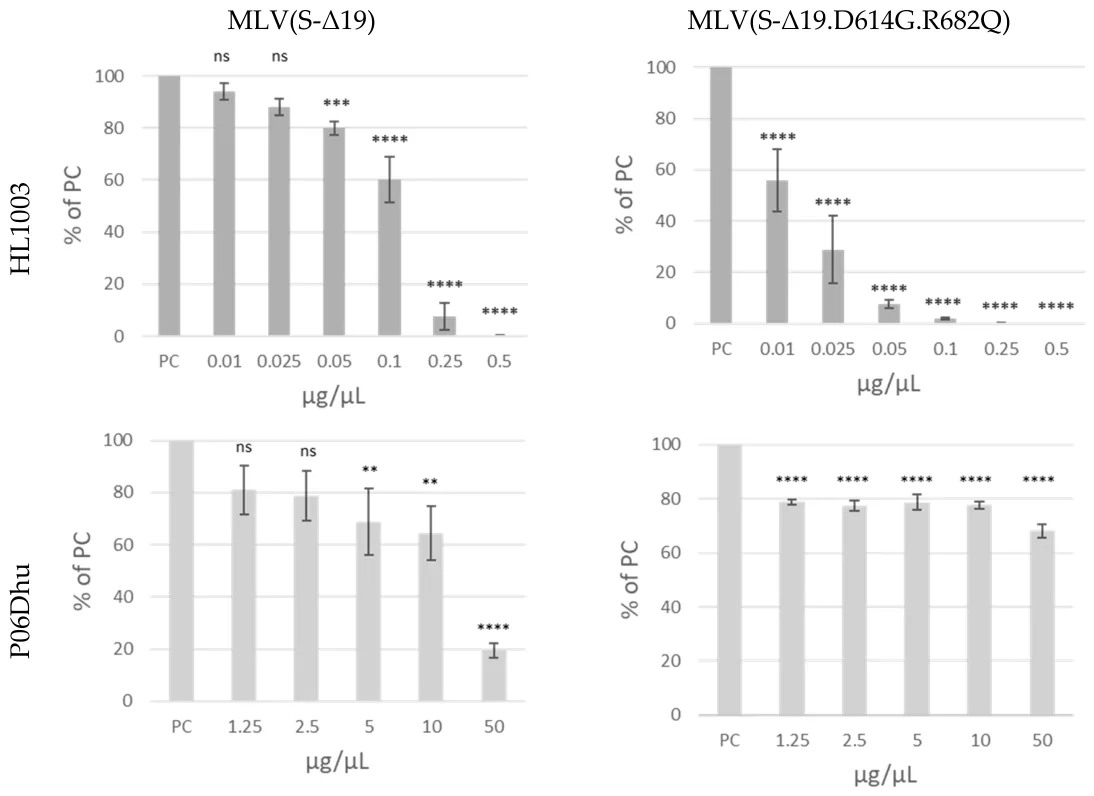
Neutralization assay using two MLV(SARS-CoV-2) pseudotype vectors and two neutralizing mAbs. Transduction efficiencies after pseudotype vector neutralization using nAbs HL1003 and P06Dhu to inhibit receptor binding. 293T/ACE2/TMPRSS2 cells were transduced using MLV(S-∆19) (**left**) and MLV(S-∆19.D614G.R682Q) (**right**) at a constant MOI but varying concentrations of one of the nAbs. A positive control (PC), transduced in the absence of nAbs, was used to normalize transduction efficiencies in percent (% of PC, y-axis). Different concentrations of nAbs were used as indicated on the x-axis. Columns show the mean results from three individual experiments. Error bars represent standard deviations. Not statistically significant (ns) = *p* > 0.05, * = *p* < 0.05, ** = *p* < 0.01, *** = *p* < 0.001, **** = *p* < 0.0001 compared to the PC.

Figure 4 visualizes the results of the VNAs for both MLV(SARS-CoV-2) pseudotypes compared to the respective PC. Ab HL1003 exhibited superior neutralizing potential, reaching full neutralization of both pseudotypes at concentrations below 0.5 µg/µL. It proved especially efficient with MLV(S-∆19.D614G.R682Q), achieving 40% reduction in gene transduction rate at only 0.01 µg/µL. The calculated IC50 values were 0.1251 with a 95% CI of 0.1037 to 0.1508 and 0.01251 with a 95% CI of 0.009963 to 0.01572, respectively.

In contrast, nAb P06Dhu did not result in any detectable neutralization within the concentration window of 0.01 to 0.5 µg/µL. Neutralization of MLV(S-Δ19) vectors did not reach 50% even with 10 µg/µL of the mAb. Yet, employing 50 µg/µL achieved more than 80% neutralization. MLV(S-∆19.D614G.R682Q) vectors appeared almost resistant against neutralization with P06Dhu, revealing only about 25% reduction in transduction efficiencies in the presence of the highest concentration of 50 µg/µL. Consequently, the determination of IC values appeared omittable.

### 3.5. Transduction Inhibition Assay Using TMPRSS2 Inhibitors

Nafamostat mesylate and Camostat mesylate are well studied TMPRS22 inhibitors, and were thus used to demonstrate the cellular assay platform’s utility for the identification and characterization and of cell-entry inhibitors. Briefly, 293T/ACE2/TMPRSS2 cells were incubated for one hour in presence or absence of the inhibitors dissolved in DMSO. Inhibitors were assessed in concentration ranges of 10 to 10,000 nM for Nafamostat and 250 to 50,000 nM for Camostat. The highest amount of DMSO used in the inhibitor dilutions was employed in target cell pre-incubation to separate potential inhibitory effects resulting from the solvent rather than the SMI. Target cells not exposed to either DMSO or any inhibitors served as PC and transduction efficiencies yielded were set as 100%. Again, light-microscopic analyses performed daily did not deliver any indications of potential cytotoxic activities mediated by the SMIs or the solvent DMSO.

**Figure 5:**
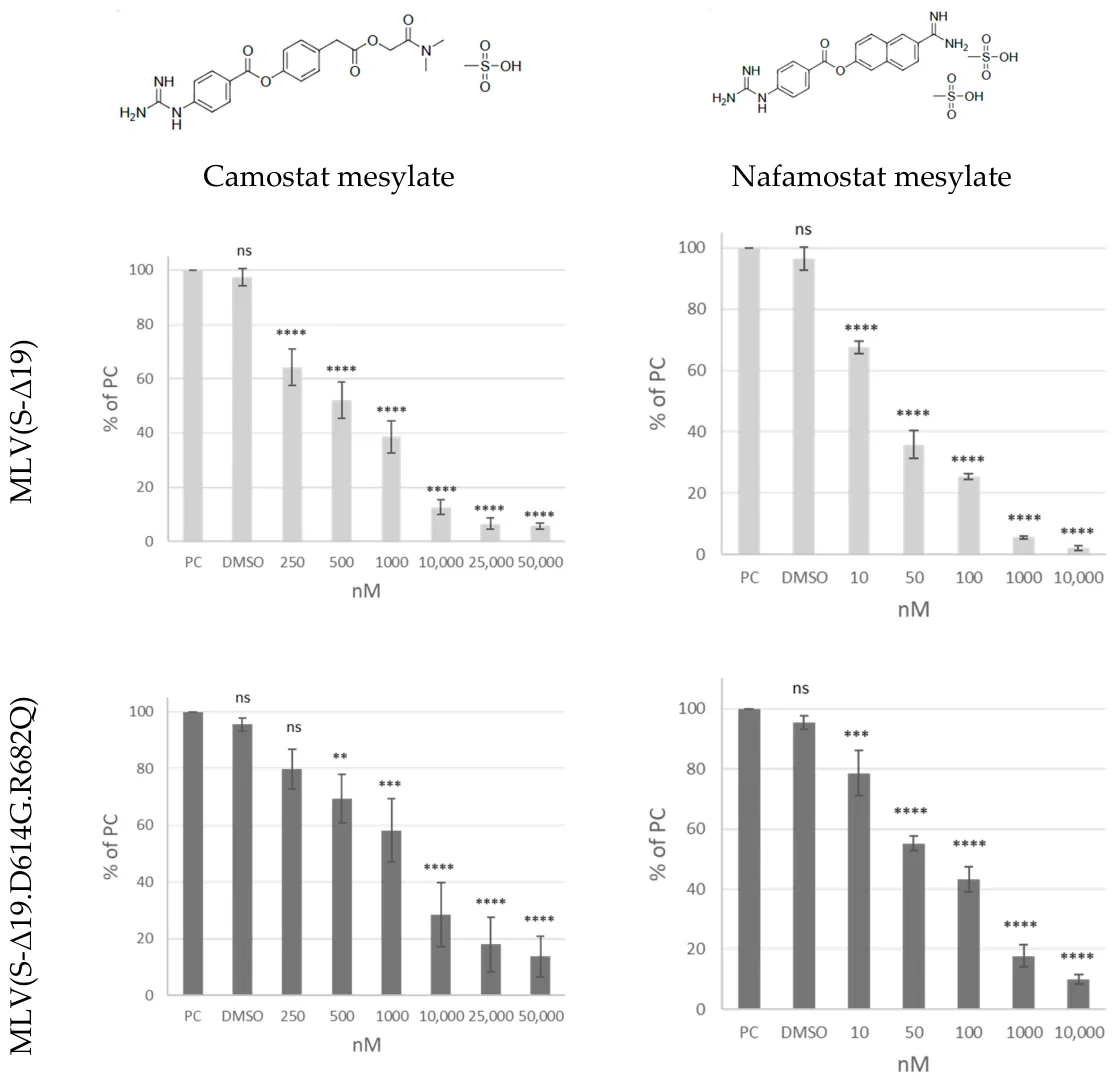
Transduction inhibition assay using MLV(SARS-CoV-2) and Camostat and Nafamostat, respectively. Transduction efficiencies after TMPRSS2 protease inhibition using Camostat mesylate and Nafamostat mesylate to block spike priming, and thus fusogenicity. Susceptible 293T/ACE2/TMPRSS2 target cells were incubated with the respective inhibitors prior to transduction using MLV(S-∆19) (**upper panel**) and MLV(S-∆19.D614G.R682Q) (**lower panel**). Dosage of inhibitors Camostat (**left**) and Nafamostat (**right**) are indicated on the x-axis. Cells were transduced with a constant MOI of MLV(SARS-CoV-2) vectors after pre-treatment with different doses of inhibitors, DMSO alone and media (PC). Transduction efficiencies were normalized to the PC (y-axis). The mean values from three independent experiments are shown and error bars indicate standard deviations. Not statistically significant (ns) = *p* > 0.05, * = *p* < 0.05, ** = *p* < 0.01, *** = *p* < 0.001, **** = *p* < 0.0001 compared to the PC.

Thawed MLV(SARS-CoV-2)-containing CFSNs were added to the target cells. Two days post-transduction, cells were analyzed using flow cytometry and transduction efficiencies were normalized against that of the PC. Results are visualized in Figure 5. Using DMSO alone, serving as a negative control, resulted in a slight reduction in transduction efficiency. The inhibitor Nafamostat was overall more potent requiring only a fifth of the dose as compared to Camostat. Nafamostat reached >95% and >90% inhibition at the highest concentration of 10,000 nM using MLV(S-∆19) and MLV(S-∆19.D614G.R682Q) vectors reaching IC50 values of 25.10 and 56.55 with 95% CIs of 21.79 to 28.92 and 43.08 to 74.24, respectively. Camostat achieved only >90% and >80% inhibition at 50,000 nM with the two vectors (IC50 of 527.7 and 1664 with 95% CIs of 406.7 to 684.5 and 554.3 to 4995), respectively.

## 4. Discussion

In this POC study, two SARS-CoV-2 spike variants were chosen as prototype viral surface proteins to demonstrate the utility of transposon vectors in the rapid establishment of SVPCs to produce pseudotype vector particles applicable in the identification—e.g., conducting a library screening campaign—and characterization of cell-entry-inhibiting small molecules and nAbs.

First, the functional expression of the two spike variants was assessed in a syncytia-formation assay. Both spike variants, pS-∆19 and pS-∆19.D614G.R682Q, transiently expressed in 293T failed to initiate cell-to-cell fusion upon co-cultivation with naive 293T cells. In alignment with previous reports, this lack of syncytia-formation in naive cells suggested very low endogenous ACE2 receptor expression [2,38,39]. In contrast and as expected, both spike proteins mediated formation of large syncytia upon co-cultivation with recombinant 293T/ACE2/TMPRSS2 cells previously shown to be highly susceptible to HIV(SARS-CoV-2) pseudotype vector-mediated gene transduction. This demonstrated the full functionality of both spike variants, namely, cell-surface expression, receptor recruitment, priming by the TMPRSS2 protease and resultant high fusogenic activity. The S1/S2 cleavage site-deficient S-∆19.D614G.R682Q spike variant’s fusogenicity was most likely restored by TMPRSS2 priming as described by Lavie and co-workers [36].

Spike protein incorporation into MLV-derived vector particles in CFSNs of both stable SVPCs was readily detected as comparable amounts of spikes per particle using S2-specific antibodies in a Western blot analysis. While variant S-∆19 was almost fully processed and detected in form of the S2 subunit, S-∆19.D614G.R682Q appeared mostly as the uncleaved, full-length spike precursor resulting from inefficient processing due to the impaired cleavage at the S1/S2 site caused by the R682Q mutation [35,36]. As previously shown in numerous reports, lower concentrations of proteins at different molecular weights were also detected, most likely representing spike processing intermediates, cleavage products, or structural variants with different N- and O-glycosylation patterns.

Vector particle titers were assessed using vector titration experiments. While naive 293T target cells revealed only very low susceptibility resulting in titers of just above 1.0 × 10^2^ TU/mL, engineered 293T/ACE2/TMPRSS2 cells allowed for the consistent detection of over 1.0 × 10^6^ TU/mL for both MLV pseudotype vectors in multiple batches of freshly harvested serum-free CFSNs. The very stable productivity of both SVPCs was thus in accordance with previous data originating from other cell lines based on comparable transposon vectors and 293-F host cells [21]. Upon freezing and thawing, vector titers of about 2.0 × 10^5^ TU/mL in the recombinant target cells were obtained for both vector particles. The approximately five-fold loss of infectivity observed was within the expected range for retrovirus-derived vectors, most importantly resulting from the degradation of the transfer vector transcripts. In conclusion, this demonstrated the high productivity of the novel SVPCs and the utility of the highly susceptible engineered 293T-derived target cells.

To show the utility of the novel platform in the identification and characterization of nAbs, MOIs of 0.1 of both vector particles were incubated with the different concentrations of nAbs HL1003 and P06Dhu followed by transduction of 293T/ACE2/TMPRSS2 cells. Flow cytometric analysis conducted two days post-transduction revealed the potent neutralization of nAb HL1003 already at comparably lower concentrations up to 0.5 µg/µL, particularly with the MLV(S-∆19.D614G.R682Q) pseudotype vectors. In contrast, nAb P06Dhu did not reveal detectable neutralization efficiencies within the range of 0.01 to 0.5 µg/µL. Consequently, the nAb concentrations were elevated to up to 50 µg/µL. Still MLV(S-Δ19) vectors were not even neutralized by 50% even when 10 µg/µL of P06Dhu were used. MLV(S-∆19.D614G.R682Q) vectors revealed an even more pronounced resistance against neutralization. Only about a fourth of neutralization of transduction was detected employing the highest concentration of 50 µg/µL. As previous studies demonstrated the utility of MLV(SARS-CoV-2) vector particles to assess the immune globulin-mediated neutralizing activity in sera from vaccinees and infected donors and showed strong correlation with live virus-based neutralization assays [20], we anticipate our approach of utilizing transposon vectors to rapidly establish high-titer SVPCs to prove valuable in the examination of polyclonal antibody samples.

Targeting the pathogen’s fusogenic surface proteins may require the ongoing and extended development of inhibitors like nAbs following the prolonged mutagenesis and immune evasion of the virus. In contrast, protease inhibitors like Camostat and Nafamostat used in this study block TMPRSS2, and thus inhibit infection mediated upon membrane fusion. Both Camostat and Nafamostat appeared to affect MLV(S-∆19.D614G.R682Q) less than the truncated wild-type, which could be attributed to the mutant variant’s presumed TMPRSS2-independence using endocytosis for cell entry, resulting from the lack of the furin cleavage of the permutated target site. These findings confirm that the loss of the S1/S2 cleavage site does not abrogate priming by TMPRSS2, since the transduction efficiency of MLV(S-∆19.D614G.R682Q) remained sensitive to both TMPRSS2 inhibitors [36]. That Nafamostat proved more potent in inhibiting transduction of MLV(SARS-CoV-2) pseudotype vectors is in line with previous reports [38,40].

The establishment of stable, continuously vector particle producing SVPCs overcomes the limited scalability of adherent VPCs generated upon transient transfection [20,41,42]. SVPCs can be easily cultivated in shaker flasks allowing for the production of larger volumes of 300 mL per 1 L flask. Moreover, such suspension cultures can easily be scaled up to much higher volumes using stirred bioreactors [21] fostering larger small-molecule compound library screens, antibody discovery campaigns and the subsequent verification and characterization of hits [21,43]. We also anticipate the panel of transposon vectors and derived cellular assay platform components to be of utility in vaccine development programs facilitating the serological examination for neutralizing antibody responses upon infection or vaccination in preclinical and clinical settings with human vaccinees [44]. Using standard molecular cloning techniques, the system is easily adaptable to further spike variants of SARS-CoV-2 and of course other viral pathogens, provided that their envelope proteins or derived variants thereof efficiently pseudotype MLV and are not cytotoxic in the host suspension cell line, and thus allow for the establishment of stable SVPCs. Such parental donor viruses include, for example, MERS-CoV, EBOV, and HIV [11,12,13,14,16,17,18,19].

## 5. Conclusions

A panel of *Sleeping Beauty*-derived transposon vectors allows for the rapid establishment of serum-free high-titer pseudotype SVPCs and highly susceptible target cells within about a month. In this POC, two SARS-CoV-2 spike variants served as prototypic viral surface proteins together with the cognate receptor ACE2 and the membrane-protease TMPRSS2 facilitating cell-entry constituting a novel recombinant cell-based assay platform. Its utility in the detection and characterization of small-molecule cell-entry inhibitors and neutralizing antibodies was demonstrated. The vector system should be easily adaptable to other donor virus spike and envelope proteins and their cognate receptors.

## Figures and Tables

**Table 1:** Primers and oligonucleotides used for molecular cloning of the spike variants and expression vector generation.

Spike_fragment 1_FOR 5′-TCGTACGTTAACATAGATAACTGATCCAGTGTGACCATGTTCGTGTTTCTGGTGCTGCT-3′
Spike_fragment 1_REV 5′-GCCCTGGTACAGCACTGCCACCTGATTGCTGGTGTTGGTGC-3′
Spike_fragment 2_FOR 5′-CAGCGGGCCAGATCTGTGGCCAGCCAGAGCATCATTG-3′
Spike_fragment 2_REV 5′-CAGCTGGCCCTCGCAGACAGCGAATTAATTCCAGCACACTGGCTAGCAGCAGCTGCCACAGCT-3′
Oligo_D614G-R682Q 5′-CTGGCACCAACACCAGCAATCAGGTGGCAGTGCTGTACCAGGGCGTGAA CTGTACCGAAGTGCCCGTGGCCATTCACGCCGATCAGCTGACACCTACAT-GGCGGGTGTACTCCACCGGCAGCAATGTGTT TCA GACCAGAGCCGGCTGT- CTGATCGGAGCCGA GCACGTGAACAATAGCTACGAGTGCGACATCCCCAT- CGGCGCTGGCATCTGTGCCAGCTACCAGACACAGACAAACAGCCCCCAGC- GGGCCAGATCT GTGGCCAGCCAGAGCATCA-3′
ACE2_FOR 5′-TAGCTAgCACCATGTCAAGCTCTTCCTGGCTCCTTCTCAG-3′
ACE2_REV 5′-GCGGCCGCCTACTAAAAGGAGGTCTGAACATCATCAGTGTTTTGGAATC-CTGG-3′
TMPRSS2_FOR 5′-TATGATATCGTACGTTAACATAGATAACTGATCCAGTGTGATGGCTTTGA-ACTCAGGGTCAC-3′
TMPRSS2_REV 5′-AACAGCTGGCCCTCGCAGACAGCGAATTAATTCCAGCACACTAGCCGT-CTGCCCTCA-3′

**Table 2:** Functional titers of freshly harvested (**A**) and freeze-thawed CFSNs (**B**) of both SVPCs. Spike variants pseudotyping MLV(SARS-CoV-2) vector particles used in transduction experiments in 293T/ACE2/TMPRSS2 target cells are indicated. I, II, and III represent three individual experiments where different harvests (batches) of freshly collected CFSNs were used in transductions experiments (**A**). In contrast, three individual aliquots 1, 2 and 3 of the same freeze-thawed CFSN batch were employed to determine the functional titer of this batch (**B**). Titers are shown in TU/mL. Standard deviations (SD) are listed on the very right.

	Spike Variant in MLV(SARS-CoV-2)	Titer (TU/mL)				
					Mean	SD
**A**		Biological replicates				
		I	II	III		
freshly harvested	S-Δ19	9.87 × 10^5^	1.65 × 10^6^	1.91 × 10^6^	**1.52 × 10^6^**	4.77 × 10^5^
	S-∆19.D614G.R682Q	2.03 × 10^6^	1.15 × 10^6^	8.35 × 10^5^	**1.34 × 10^6^**	6.22 × 10^5^
**B**		Technical replicates				
		1	2	3		
freeze-thawed	S-Δ19	2.22 × 10^5^	2.09 × 10^5^	1.82 × 10^5^	**2.04 × 10^5^**	2.05 × 10^4^
	S-∆19.D614G.R682Q	2.13 × 10^5^	1.71 × 10^5^	1.95 × 10^5^	**1.93 × 10^5^**	2.09 × 10^4^

## Data Availability

The original contributions presented in this study are included in the article. Further inquiries can be directed to the corresponding author.

## Abbreviations

The following abbreviations are used in this manuscript:

ACE2	angiotensin-converting enzyme 2
BSA	bovine serum albumin
BSL	bio-safety level
CI	confidence interval
CFSN	cell-free supernatant
DMEM	Dulbecco’s Modified Eagle Medium
EBOV	Ebola virus
EGFP	enhanced green fluorescent protein
HEK	human embryonic kidney
hygR	hygromycin B resistance gene
IC50	half-maximal inhibitory concentrations
In	synthetic intron
IRES	internal ribosome entry site
IVA/B	influenza virus A and B
kDa	kilodalton
mAbs	monoclonal antibodies
nAbs	neutralizing antibodies
MLV	murine leukemia virus
MOI	multiplicity of infection
neoR	neomycin resistance gene
p(A)	polyadenylation signal
PC	positive control
P_CMV_	CMV promoter/enhancer element
PEI	polyethylenimine
POC	proof-of-concept
puroR	puromycin resistance gene
RABV	rabies virus
RBD	receptor binding domain
RSV	respiratory syncytial virus
S	SARS-CoV-2 spike protein
SD	standard deviation
SVPC	suspension vector producer cells
TBS-T	Tris-buffered saline with Tween-20
TIR	terminal inverted repeats
TMPRSS2	transmembrane serine protease 2
TU/mL	transducing units per milliliter
VNA	virus-neutralization assay
ZIKV	zika virus
